# Harnessing the power of hashtags: Temporal pattern mining and storyline construction for event evolution on social media

**DOI:** 10.1371/journal.pone.0327596

**Published:** 2025-08-28

**Authors:** Jinbao Song, Yu He, Xingyu Zhang, Nuo Xu, Guangtao Fu

**Affiliations:** 1 State Key Laboratory of Media Convergence and Communication, Communication University of China, Beijing, P.R.China; 2 School of Information and Communication Engineering, Communication University of China, Beijing, P.R.China; 3 School of Data Science and Media Intelligence, Communication University of China, Beijing, P.R.China; 4 Academy of Broadcasting Science, NRTA, China; Roma Tre University: Universita degli Studi Roma Tre, ITALY

## Abstract

With the rapid development and wide application of social media, Weibo, as one of the major social media platforms in China, plays an important role in connecting users with information. However, the huge amount of Weibo data poses challenges for effective analysis and understanding. Timeline construction is critical for understanding event progression, enabling stakeholders to track public opinion shifts, identify critical phases of event development, and formulate timely interventions. This paper proposes a framework to systematically model event evolution by analyzing temporal patterns and semantic correlations of hashtags. We first adopt a temporal feature extraction method to capture the temporal information of Weibo posting time. Then, the correlation between topic tags is considered comprehensively by combining the temporal information and the similarity calculation method of topic tags. Finally, a timeline-based topic merging algorithm is proposed to construct a clear and orderly event story line. Meanwhile, this paper also introduces the RoMLP-AttNet model, which significantly improves the classification recall and precision in the Weibo event classification task by using the topic posting sequences as the background data for assisting event detection. Using the "Japanese nuclear effluent" event as an example, the story line construction method proposed in this paper generated a clear and complete story line. Experimental results demonstrate that the RoMLP-AttNet model proposed in this paper achieved an average increase of 16.73% in recall rate, 15.8% in precision, and 17.38% in F1 score.

## Introduction

In the contemporary landscape, characterised by the heightened development and widespread utilisation of social media, Weibo stands as one of China’s foremost social media platforms, playing a pivotal role in connecting users with information. The vast corpus of Weibo data contains a wealth of social events and topics, including emergent incidents, societal hotspots, and focal points of public opinion. Timely and accurate analysis of these events has the potential to facilitate governmental, media, and corporate entities in comprehending societal sentiments, forecasting trends, and formulating corresponding strategies. For instance, during the “Japanese nuclear effluent" event, fragmented discussions with hashtags like “#JapanNuclearWastewater" and “#FukushimaDischarge" emerged sporadically. Manual analysis failed to capture their temporal interdependencies, while unsupervised methods produced incoherent clusters. However, the high-dimensional, heterogeneous, and temporally dynamic nature of Weibo data, coupled with the presence of information noise and rumours, poses significant challenges to the coherent analysis of massive Weibo datasets. Conventional manual analysis and supervised learning approaches encounter difficulties when handling such datasets, while unsupervised methods lack effective guidance and accuracy. Consequently, unresolved issues persist. Given these practical challenges, there is a pressing need to explore novel solutions.

Distinct from other global social media platforms such as Twitter, Weibo exhibits several unique characteristics in information dissemination. Firstly, Weibo’s high degree of real-time interaction enables users to rapidly share and access information as events unfold. Secondly, its short-text format encourages concise and efficient communication. Notably, Weibo hashtags are often spontaneously created around specific events, which facilitates rapid event aggregation and the formation of event-centric communities. This event-driven and organic hashtag usage reflects the platform’s unique cultural and social dynamics. Hashtags on Weibo serve as subjective expressions of user engagement with events. They not only encapsulate abundant information but also reflect the extent of attention and discourse within user communities regarding specific events. The intrinsic nature of hashtag usage renders it a unique and potent indicator of events. When an event garners widespread attention, users often participate in discussions by appending relevant hashtags to their Weibo posts, thereby forming a virtual, instantaneous event community. Consequently, the aggregation of Weibo hashtags not only serves as a medium for collective expression of events by users but also functions as a crucial metric for gauging the impact of events and the sentiment of users.

This paper presents a detailed examination of the evolution of events on the Chinese social media platform Weibo. It employs the analysis of hashtags in Weibo data to identify patterns in the growth and development of events on the platform. Specifically, this study proposes a framework that integrates temporal features and semantic correlations of hashtags to address the challenges of extracting coherent event trajectories from high-noise, fragmented Weibo data. This approach enables fine-grained modeling of event timelines and underlying semantic structures. The objective is to gain a deeper understanding of the structural changes that have occurred over time.

Our main contributions are threefold. First, we propose an innovative approach to extract temporal hashtag release sequences as features, enabling comprehensive similarity calculations by integrating semantic and temporal correlations. This method addresses the limitations of existing techniques that either neglect temporal dynamics or oversimplify semantic relationships. Second, we design a timeline-based merging algorithm that hierarchically clusters key hashtags into coherent storylines. By leveraging both chronological order and semantic similarity, this algorithm constructs event evolution paths that reflect real-world interdependencies. Finally, we introduce the RoMLP-AttNet model, which leverages hashtag posting sequences as auxiliary data to improve event classification accuracy by at least 15.8%. This model combines deep semantic analysis with explicit temporal modeling, outperforming traditional methods in handling noisy and fragmented social media data.

## Related work

### Event detection

Event detection serves as the foundation for extracting open-domain events, typically achieved through the extraction of event keywords and clustering of similar events. In contrast to news reports, Weibo text is shorter, less coherent, difficult to amalgamate into documents, and often reflects richer emotions and viewpoints. Event representations in Weibo are more fragmented, characterised by immediacy and high noise levels, yet Weibo hashtags offer new possibilities for extracting event contexts. This paper therefore focuses on the current research status of event detection in social media.

Event detection tasks can be divided into methods based on keyword relevance, topic modelling, incremental clustering, and hybrid approaches [1]. Methods for extracting Weibo event keywords include frequency-based statistics [2], TF-IDF [3,4,8], and text clustering [6]. While frequency-based statistics and TF-IDF methods are straightforward, they overlook semantic information of words, are susceptible to common word interference, and struggle to capture semantic features of event keywords. While text clustering methods can extract representative words, they require high-quality clustering algorithms and results. This necessitates a balance between diversity and representativeness. Some methods may tend to select highly representative but highly repetitive words, neglecting more semantic information.

Methods based on topic modelling are employed to identify themes and topics within textfor event detection. Representative models include Probabilistic Latent Semantic Analysis (PLSA) [7], Latent Dirichlet Allocation (LDA) [8,9], but face challenges in social media analysis: the sparse and short nature of Weibo texts exacerbates high dimensionality and sparsity issues. In a related study, Shi et al. [10] proposed a hybrid RNN-topic model to capture word relationships and mitigate sparsity. However, topic-based models tend to favour high-frequency feature words, overlooking contextual nuances and word dependencies.

In contrast, incremental clustering [11,12] dynamically processes streaming data through algorithms like K-means and DBSCAN. Texts are vectorized before clustering, where vector quality critically impacts results This method of event detection is achieved by especially crucial for noisy Weibo texts. New advancements in incremental clustering include community detection algorithms, which leverage social network structures to dynamically cluster real-time Twitter data into event communities. For example, Singh et al. [54] combined graph-based community detection with temporal features to identify emerging events in streaming data. Additionally, Guo et al. proposed a hybrid framework of graph contrastive learning and reinforced incremental clustering (HCRC) [55], which uses contrastive learning to capture semantic-contextual features and adaptive clustering to handle concept drift in noisy Weibo data.

For hybrid approaches, recent studies integrate deep learning with clustering or spatio-temporal analysis. For instance, Afyouni et al. developed Deep-Eware [52], a model combining CNN-LSTM for text feature extraction with hierarchical density-based clustering to detect spatio-temporal events in social media. Kolajo et al. proposed SMAFED [53], which enhances semantic analysis of noisy terms via knowledge graph integration and incremental clustering based on semantic histograms, improving event detection accuracy in real-time streams.

In recent years, deep learning techniques have been widely applied in event detection. For example, Zhou et al. [14] combined CNN and RNN methods, effectively capturing local features and semantic information of text, thereby improving classification performance. Although deep learning methods [15–18] have made significant progress in improving accuracy and reducing redundancy in feature design, it is worth noting that these methods often face challenges in practical applications, such as they incur high training costs, requiring substantial time and computational resources. Furthermore, once trained, model parameters cannot be easily modified. This aligns with our observations in experimental settings, where large-scale data annotation and extended training periods were consistently required.

In conclusion, methods based on keyword relevance focus on word frequency features but neglect semantic information, methods based on topic modelling can identify themes but ignore contextual relationships, and deep learning methods can enhance accuracy but are costly and difficult to apply to real-time updates in social networks. Consequently, in the current context, methods based on incremental clustering are more suitable, as they can handle new text data in real-time, overcome the limitations of other methods, require fewer labelled data, reduce costs, and demonstrate better adaptability and feasibility in handling real-time updates in social networks.

### Storyline construction

Given the vastness of social media data and the abundance of noise within it, obtaining clear storylines directly from social platforms is evidently a highly challenging task. Manual curation of storylines not only demands significant time and effort but also lacks scalability. Therefore, automatic construction of storylines emerges as a crucial task in event context analysis. Zhao et al. [19] distinguish between four principal approaches to the construction of storylines: association-based, feature modelling-based, propagation model-based, and timeline-based methods.

Association-based methods include two categories: similarity-based methods [22,23,26,27] link events via semantic or temporal metrics, while clustering-based methods [28–31] organize events hierarchically. Feature modelling-based approaches analyze feature space distances, including Bayesian models [32–34], Biterm topic models [35], non-negative matrix factorisation [36,37], and neural networks [38]. Propagation models use [39–42] or graph structures [43]. Timeline-based approaches chronologically order events via optimization [44] or ML algorithms [45–47]. Extensions to multimodal data [48] and document temporal expressions [49]) are also explored.

However, Weibo’s short texts and noise limit association-based methods’ accuracy. Propagation models oversimplify real-world complexity, while timeline approaches produce linear narratives that ignore event interdependencies. Therefore, feature modelling as BERT-based semantic extraction with temporal features better suits Weibo data. Combined with timeline similarity metrics, this enables coherent storyline construction reflecting event evolution.

## Introduction to relevant concepts and datasets

A story line construction method based on temporal information and topic tag similarity is proposed, which can generate a clear and complete event story line.

### Introduction to related concepts

**Fig 1:**
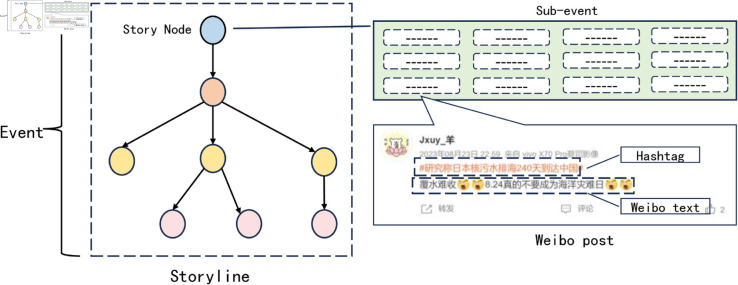
Relevant conceptual relationship diagrams.

Given the abundance of statements pertaining to “events", “sub-event", “reports", and other related topics, it is essential to provide clear definitions for all key terms in order to facilitate a comprehensive understanding of the content of this paper. In light of relevant literature and specific research, this paper will offer precise definitions for all key terms, ensuring a coherent and accurate interpretation of the subject matter. The following Fig 1 is relevant to the conceptual framework of this study.

- Event: A compendium of all sub-events pertaining to a specific incident. For example, all Weibo posts related to the term “Japan nuclear wastewater" are considered part of the “Japan nuclear wastewater" event.
- Sub-event: An occurrence at a specific time or place. In comparison to the definition of “event," “sub-event" has a narrower scope and provides a more specific description of the occurrence. To illustrate, the event “Japan nuclear wastewater" is a major occurrence, whereas the sub-event “Japan starting the discharge of nuclear wastewater on the 24th" is one of its constituent parts.
- Story line: A tree-like structure, comprising story nodes, is employed to represent the relationships between nodes and to illustrate the overall trend of event development. In contrast to events, story lines are more concerned with the interconnections between sub-events.
- Story Node: The nodes of the story line represent sub-events. To illustrate, the sub-event “Japan started nuclear sewage discharge from 24th" is a compilation of all Weibo posts pertaining to this event. In turn, “Japan started nuclear sewage discharge from 24th" itself represents the sub-event as a node in the story line.
- Weibo post: A Weibo post is a concise statement on a particular topic. The set of all Weibo posts related to a specific event constitutes a sub-event.
- Weibo text: A text that is intended to convey a specific description or to express a particular point of view within the context of a narrative.
- Hashtag: A Weibo hashtag is a sequence of two ‘#’ signs, which may be accompanied by a sentence or phrase. The addition of a hashtag to a post allows the publisher to indicate the subject matter of the story, while the Weibo platform facilitates the aggregation of stories on the same subject for convenient browsing. It is important to note that not every story contains a hashtag; some only have the body of the post. As most hashtags encapsulate the subject matter, this paper posits that high-quality hashtags can be employed as sub-event representations, i.e., story nodes, in the construction of the story sequence.

### Introduction to the datasets

**Fig 2:**
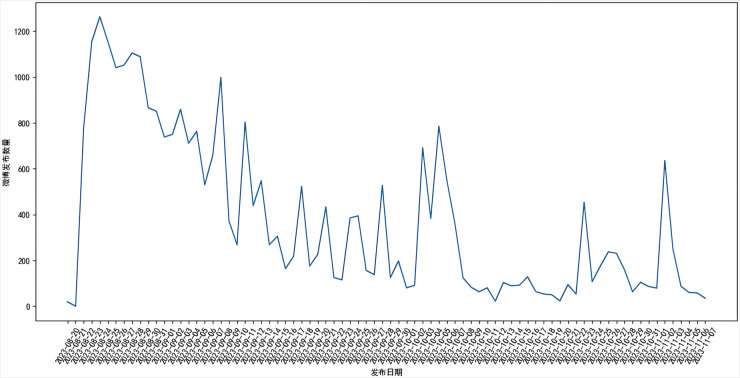
Graph of number of Weibo posts versus date.

This paper selects the “Japanese nuclear sewage" event on the Sina Weibo platform as the research object, from 20 August 2023 onwards, using “Japanese nuclear sewage" as the keyword, every 24 hours incremental crawling Weibo data related to the event, crawling the number of obtained Weibos with the date changes. The variation of the number of Weibos with the date is shown in Fig 2.

The crawled fields cover all aspects of the report, including but not limited to release time, text content, user information, etc., as shown in Table 1. As of 7 November 2023, this paper has successfully obtained 78,882 pieces of raw data, providing a sufficient and detailed data base for an in-depth study of the event. The Weibo data used in this study was collected through Sina Weibo’s public API in compliance with the platform’s terms of service. All data were anonymized to protect user privacy. No private or sensitive information was accessed or stored.

**Table 1:** Key fields and meanings of Weibo.

Field	Meaning
UserName	Username of the Weibo publisher
CreatAt	Time of Weibo publication
Authentication	Weibo VIP authentication
Content	Text content of the Weibo
Hashtag	Hashtag labels extracted from Weibo text
RepostNum	Number of reposts received by the Weibo
CommentNum	Number of comments received by the Weibo
LikeNum	Number of likes received by the Weibo
Comment	Text content of the Weibo comments
CommentMark	Comment tag
CommentTime	Time of comment publication
CommentLike	Number of likes received by the comment
CommentName	Username of the comment publisher

We perform the following preprocessing operations on the data:

(1) Extracting topic tags from Weibo text using regular expressions;
(2) Cleaning the text to remove all content irrelevant information in the text, such as emoticons, web links, @ symbols and so on;
(3) Deleting the data whose text is empty after cleaning;
(4) Data de-duplication, i.e., removing duplicate data.

#### Story lines dataset.

For the task of event story line analysis, the focus is on extracting key events and constructing the story line from the massive amount of Weibo text, the fields that need to be used in this paper are the Weibo username, the content of the Weibo text, the hashtag label, the Weibo posting time, as well as the number of Weibo retweets, comments, and likes, and the remaining Weibo comment related data is not needed. Therefore , the cleaned dataset is further processed including the following steps:

(1) Remove the fields related to the comment of the Weibo text, including comment content, comment tag, comment username, comment posting time, and the number of comments being liked;
(2) Check whether the content of Weibos contains Hashtag keywords, and remove the Weibos whose Hashtag is completely unrelated to the body text;
(3) Remove the data without Hashtag;
(4) Data de-weighting.

After the above processing, 9311 experimental data and 3789 Weibo Hashtag tags were obtained.

#### Classified datasets.

The goal of the classification is to categorise those Weiboging reports that cannot be clearly classified into the sub-event to which they belong. These reports that cannot be directly and clearly classified into the sub-event to which they belong fall into two main categories:the report does not contain a hashtag and the hashtag in the report is not in the set of candidate hashtags.

By classifying these reports, we can identify the Weibo reports within each sub-event, and ultimately analyse these Weibos for group sentiment influence. The labelled data is divided into the following two cases:

(1) For those Weibo posts whose text directly contains the node Hashtag, this paper uses the Hashtag as a tag;
(2) For those Weibos whose text contains a Hashtag that is a candidate Hashtag, this paper takes the node Hashtag formed by the final merging of that Hashtag as a label. For example, “Japan started the second round of sea discharge" and “Japan’s second round of nuclear contaminated water discharge will start from 5th October" are merged to form the node “Japan’s second round of nuclear contaminated water discharge will start from 5th October.” In this case, all Weibo texts containing the phrase “Japan’s second round of sea discharge will begin" will be tagged with the phrase “Japan’s second round of nuclear contaminated water discharge will begin on 5 October", and will become labelled data.

Finally, we take the storyline as a reference, and use the story nodes therein as data labels, which are screened from the original dataset, and finally get 7122 labelled data.

## Method

**Fig 3:**
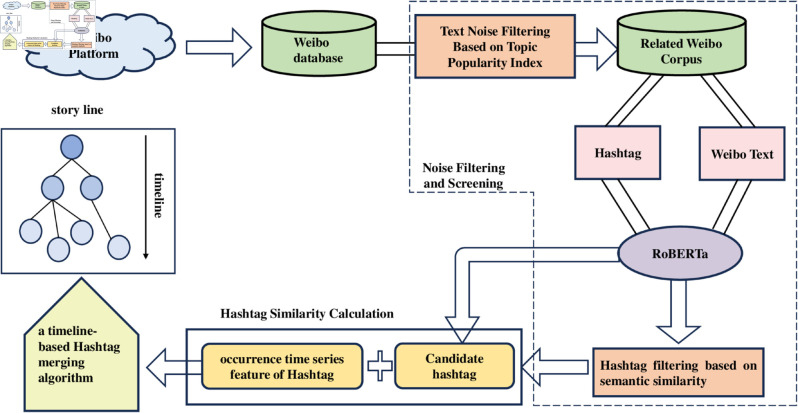
Research framework.

The following section presents an algorithm framework for automatic event story line construction based on RBT3, as illustrated in Fig 3. The algorithm framework is comprised of three principal components: noise filtering and screening, hashtag similarity calculation, and story line construction. Firstly, this article presents a novel approach, namely “text noise filtering based on topic popularity index," which effectively filters out irrelevant text in Weibo, thereby ensuring that the obtained Weibo corpus is of higher quality. Subsequently, this article employs the RBT3 model to compute vector representations of hashtags and text content in each Weibo. This approach involves the use of a tag filtering strategy based on semantic similarity analysis, which allows for the removal of irrelevant hashtags from the corpus. The remaining hashtags are then retained as candidate nodes for the event story line. Subsequently, this article extracts the release time sequence of hashtags on the timeline and utilises it as a time feature. This article employs a comprehensive approach to calculating the similarity between hashtags, integrating the feature representations of hashtags. Finally, a timeline-based hashtag merging is proposed. The algorithm hierarchically clusters key hashtags, thereby forming a clear event story line.

### Noise filtering and screening

#### Text noise filtering based on topic popularity index.

This article collates a number of Weibo posts on a range of topics, but these are interspersed with a considerable amount of irrelevant material. The noise may include content that is irrelevant to the topic, unrelated, of low quality, or false, making it difficult to gain a deep understanding and analyse the topics. To address this issue, this article proposes a noise filtering and screening method based on the topic popularity index.

The core of this method is to calculate the topic popularity index (hot_index) for each Weibo post, as shown in Equation 1. This combines social interaction information such as reposts, comments, and likes to quantify the relevance of each post to the topic. This method can more accurately assess the relevance of texts and distinguish texts closely related to the topic, thereby eliminating noise information that is unrelated to the topic.

hot_index=α×RepostNum+β×CommentNum+γ×LikeNum (1)

The formula is based on the weighting coefficients *α*, *β*, and *γ*, which sum to 1. These coefficients are crucial as they reflect the varying degrees of influence each type of social interaction has on a topic’s perceived popularity and reach. The selection of these parameters is based on the understanding that different interaction types signify different levels of user engagement and content dissemination. Reposts often indicate a strong endorsement of the content and significantly broaden its visibility. Comments represent active user engagement and discussion around the topic. Likes are a more passive form of approval but still contribute to the overall visibility and positive reception of the content. This exhaustively reflects the popularity of each text within the topic. By sorting according to the topic popularity index, this article can focus on texts that attract greater attention, thereby reducing the impact of irrelevant information. In general, the text noise filtering based on topic popularity index represents an innovative solution to the problem of dealing with a large amount of noisy text. This is achieved through in-depth information analysis and consideration of social interaction data. This method allows for more accurate identification of pertinent information related to the topic, thereby enhancing the quality and credibility of topic analysis.

#### Hashtag filtering based on semantic similarity.

In the process of processing Weibo content, publishers may arbitrarily add topic tags to Weibo texts in order to increase exposure and attract more readers, given the high uncertainty of the content. However, this practice results in the proliferation of a considerable number of tags in the Weibo dataset that are unrelated to the keyword events, thus necessitating the implementation of an effective filtering process. In order to accurately filter out information that is not directly related to specific events, this article proposes a hashtag filtering strategy based on semantic similarity analysis. The specific steps are as follows:

(1) The pre-trained word vector model RBT3 is employed to calculate the vector representations H and P of hashtags and text in each Weibo text, respectively. Formulas are as follows:
  H=RBT3(HashTag) (2)
  P=RBT3(Content) (3)
(2) The semantic similarity between the hashtag vector H and the text vector P is to be calculated using cosine similarity, according to the following formula:
  Sim(H,P)=cosine_similarity(H,P) (4)
(3) Setting the relevance threshold and filtering the text with similarity below the threshold.

The purpose of this method is to effectively reduce the noise interference of irrelevant hashtags and ensure that the extracted set of hashtags is more accurate and representative. This step allows for a more accurate capture of the key hashtags related to the event as the story line candidate node, thereby providing a more reliable data base for the subsequent event story line analysis.

### Hashtag similarity calculation

Due to the existence of disparate representations of the same event on Weibo, such as ‘#Japan Nuclear Wastewater’ and ‘#Japan Nuclear Sewage’, it is necessary to enhance the consistency of the hashtags and the overall data quality. To address the issue of the diversity of hashtag representations in Weibo data, this paper employs a multi-dimensional synthesis approach. This approach combines text feature extraction, time series analysis, and semantic similarity computation. Its objective is to process and merge similar or duplicate hashtags in a more comprehensive manner, thereby improving the consistency and quality of the data.

First, by concatenating the Hashtag feature vector H and the text feature vector P for each post containing the hashtag, and then averaging the accumulated concatenated vectors, this paper obtains the feature representation of the Hashtag, as shown in Equation 5. Here, N represents the number of Weibo posts containing the respective Hashtag. The concatenation of the Hashtag and text feature vectors is designed to integrate both the explicit meaning of the tag and the contextual information from the associated post, thereby providing a richer and more informative representation. This feature representation not only encapsulates the semantic information of the tags themselves but also incorporates information about the events represented by the tags. By concatenating hashtags and posts, this integrated approach serves to enrich the semantic context of hashtags by linking them to their original conversational threads and to enable the temporal analysis of their co-occurrence patterns. This, in turn, allows for the tracking of how hashtags evolve and interact over time, ultimately providing deeper insights into community dynamics.

$$tag\_embedding=\frac{1}{N}\sum_{i=1}^{N}\text{concat}(H_{i},P_{i})$$ (5)

Subsequently, this paper employs the cosine similarity calculation method to compute these integrated features with the objective of identifying tags exhibiting high similarity for merging. In this step, the semantic similarity between the hashtags, designated text_similarity, is computed initially, as delineated in Equation 6.

$$text\_similarity=\text{cosine\_similarity}(tag\_embedding_{i},tag\_embedding_{j})$$ (6)

**Fig 4:**
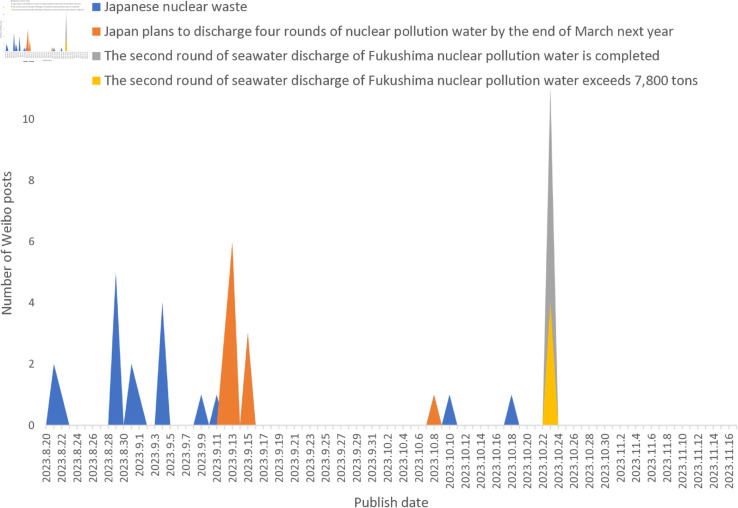
Hashtag post number over time.

We introduce the occurrence time series feature (time_feature) of Hashtag to calculate the temporal similarity (time_similarity, see Equation 7) of Hashtag, which indicates the number of occurrences of a tag and its distribution on the time axis. Unlike text similarity, time_similarity is concerned with the distribution and occurrence pattern of the tag on the timeline. Fig 4, where each line represents a different hashtag, illustrates the temporal evolution of the number of posts for four hashtags: ‘Japanese nuclear waste’, ‘Japan plans to discharge four rounds of nuclear pollution water by the end of March next year’, ‘The second round of seawater discharge of Fukushima nuclear pollution water is completed’, and ‘The second round of seawater discharge of Fukushima nuclear pollution water exceeds 7,800 tons’.

Looking at the timeline, the hashtag “Japan plans to discharge four rounds of nuclear pollution water by the end of March next year" was prominently present on September 13, 2023, and September 16, 2023. However, “The second round of seawater discharge of Fukushima nuclear pollution water is completed" and “The second round of seawater discharge of Fukushima nuclear pollution water exceeds 7,800 tons" only appeared on October 24, 2023. While there are significant differences in the content of these hashtags, “The second round of seawater discharge of Fukushima nuclear pollution water is completed" and “The second round of seawater discharge of Fukushima nuclear pollution water exceeds 7,800 tons" share high similarity. This suggests that tags with high similarity also demonstrate similar patterns in their appearance over time. Thus, leveraging time features allows for the analysis of tag behavior and trends along the temporal axis, enabling better management and merging of similar or duplicate tags.

$$time\_similarity=\text{cosine\_similarity}(time\_feature_{i},time\_feature_{j})$$ (7)

Finally, to comprehensively assess the similarity between two tags, this paper combines text similarity and time similarity. Referring to Equation 8, weight parameters a and b are introduced to adjust the emphasis on text and time features according to practical needs. By adjusting these weight parameters, the calculation of both similarities can be flexibly balanced, thereby more accurately determining the similarity between tags. This integrated approach provides a flexible and comprehensive way to evaluate the correlation between tags, facilitating better handling and merging of similar or duplicate tags.

similarity=a×text_similarity+b×time_similarity (8)

By setting parameters a and b, this paper effectively calculates the similarity between hashtags, providing a solid foundation for deeper understanding of their intrinsic relationships. Subsequently, a threshold is set to filter out hashtags with higher similarity for merging. By adjusting this threshold, the paper can flexibly control the strictness of the merging operation to better meet various research needs.

### Construction of story line

Once the Hashtag nodes have been acquired, the primary objective is to ascertain the relationship between the nodes in order to construct the event story line. This paper introduces a timeline-based Hashtag merging algorithm, which employs the sequential nature of the timeline to merge Hashtag nodes with a high degree of similarity. This process is intended to construct an ordered and clear event story line. The algorithmic process is detailed in Algorithm 1.

**Algorithm 1: Timeline-based Hashtag merging algorithm.**

**Input:Hashtag node set**

**Output:story line directed graph**

**1 G← create_di_graph();**

**2 added_nodes ← set();**

**3 for idx, d in enumerate(tag cluster) do**

**4**  **original array ← tag cluster[d];**

**5**  **result array ← filter zeros(original array);**

**6**  **cluster ← remove duplicates(result array);**

**7**  **if idx == 0 then**

**8**   **for c in cluster do**

**9**    **add node to graph(G, tags[c-1]);**

**10**    **added nodes.add(c);**

**11**   **end**

**12 end**

**13 else**

**14**   **for c in cluster do**

**15**    **if c not in added nodes then**

**16**     **add node to graph(G, tags[c-1]);**

**17**     **max_simi_node ← find max_**

**similarity(added_nodes,tags[c-1]);**

**18**     **add_edge_to_graph(G, tags[max_**

**similarity(simi_node-1],tags[c-1]);**

**19**     **added_nodes.add(c);**

**20**    **end**

**21**   **end**

**22**  **end**

**23 end**

**24 return G;**

Firstly, this paper identifies the earliest node in the timeline associated with the Hashtag cluster as the starting point of the narrative. This node may be regarded as the root node of the entire event story line. Subsequently, in the order of the timeline, this paper compares the similarity between the subsequent node and the existing nodes in the current story line one by one, and selects the node with the highest similarity as the parent node, incorporating it into the corresponding branch in an orderly manner. This iterative process is repeated until each node is successfully integrated into the entire story line. The algorithm’s distinctive feature is its capacity to effectively establish associations between hashtag nodes in the temporal dimension. This implies that this paper is capable of organising the related nodes into a comprehensive sequence of events, based on a chronological order.

Through this algorithm, this paper achieves the hierarchical organisation of Hashtag nodes, which makes the development of the event present a clear and orderly structure. Compared with the traditional story line construction method, this algorithm is outstanding in that it makes full use of temporal information, so that the timeline of the event can run through the entire process of constructing the line. This unique design provides this paper with an in-depth understanding of the evolution of Weibo events, so that the construction of the story line is no longer a simple connection of nodes, but a closer presentation of the real situation of the event. Therefore, this algorithm provides this paper with an insightful and orderly method for presenting and understanding the dynamic development of events in Weibo.

### Weibo sub-event detection and classification

In the preceding section, a tree diagram was constructed in order to provide an in-depth understanding of the evolution of the topic’s content. The next crucial step is to specify the coverage corresponding to each sub-event. We propose a time series-based RoMLP-AttNet model for Weibo event detection. The model not only delves deeper into the semantic content of Weibo text, but also employs the temporal information of hashtag posting sequences in a strategic manner. By leveraging the hashtag release sequence as background data to enhance event detection, the model significantly enhances the accuracy of event detection.

**Fig 5:**
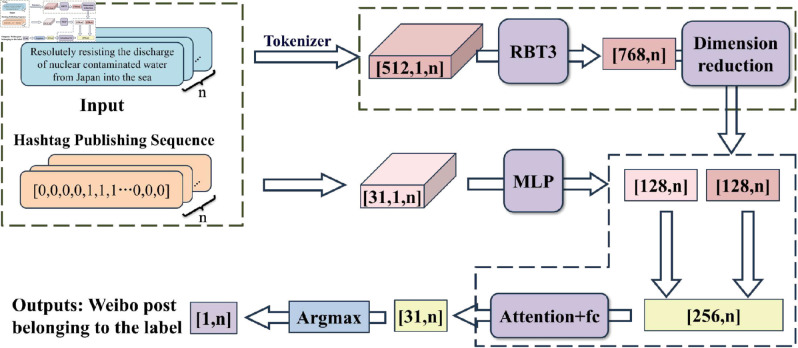
RoMLP-AttNet modelling framework.

The RoMLP-AttNet model is based on a time series framework, as illustrated in Fig 5. Unlike traditional models TextBiLSTM+Attention, which only contain semantic feature extraction paths, RoMLP-AttNet innovatively introduces a temporal feature modeling module to address the dynamic nature of events. Its core is the deep feature extraction and fusion of Weibo text and release sequence. We employs the RBT3 model to achieve the precise extraction of deep semantic features of Weibo text, thereby ensuring a comprehensive grasp of the original information. Subsequently, the extracted features are optimised by a dimensionality reduction network, which removes redundant information and makes them more representative. In order to capture the temporal dynamic information of the event in a more comprehensive manner, this paper employs a multilayer perceptron (MLP) to extract the features of the hashtag posting sequence within the context of the Weibo posting time. This temporal feature path (MLP) complements the semantic feature path (RBT3), forming a dual-branch architecture for joint modeling of “what happened" and “when it happened". Furthermore, the text features and time series features are combined in an organic manner in order to ensure that the event characteristics are reflected from multiple perspectives. Finally, the attention mechanism and the fully connected layer are employed to weight and fuse the various features. This is followed by the application of a classifier to determine the event node to which the Weibo post belongs.

#### Feature extraction.

1. Semantic feature extraction

The RBT3 model is employed in this study for extracting features from Weibo texts, enabling in-depth exploration of their connotations and precise capture of latent semantics. By comprehensively grasping the original information, the RBT3 model successfully extracts highly representative features from Weibo texts, endowing them with richer information expression. Its uniqueness lies in the profound analysis of Weibo texts, not only focusing on the superficial meaning of individual words but also emphasizing the semantic correlations between contexts, thereby presenting a more comprehensive and accurate text representation.

tokens=Tokenizer(text,padding=512,return_tensors=“pt”) (9)

$$text\_embedding^{\prime }=RBT3(tokens)$$ (10)

Given the distinctive attributes of Weibo texts, which are typically defined by their concise nature and limited information content. The objective of this study is to examine the branching events of a topic in Weibos, which may exhibit high similarity among them. In light of the aforementioned considerations, this paper notes that the 768 dimensions of the traditional BERT output appear to be excessively lengthy for Weibo texts, rendering it challenging to adequately capture key features within the constrained text length. Firstly, the high dimensionality of the traditional BERT output may result in the duplication of information and the potential for overfitting during the training process, particularly when dealing with contexts such as Weibo texts where the information is limited and relatively noisy. Secondly, the necessity for a high-dimensional representation necessitates the utilisation of greater computational resources, which may result in efficiency issues when dealing with large quantities of Weibo data. Consequently, we have opted to utilise a dimensionality reduction network, where ’Reduce’ refers to a process such as passing the high-dimensional embedding through a linear layer or applying a pooling operation to decrease its dimensionality, in order to downscale the features, thereby enabling the utilisation of the limited textual information in a more efficient manner.

$$text\_embedding=Reduce(text\_embedding^{\prime })$$ (11)

2. Temporal feature extraction

In order to capture the temporal information of Weibos more comprehensively, in the process of constructing the model, this paper introduces a key temporal feature, i.e., the set of Weibo postings at each time node. Specifically, this paper assigns a corresponding timing label to each Weibo post by determining whether the release time of the Weibo post falls within the defined time period of each sub-event. “1" indicates that a sub-event occurs at that time, and “0" indicates that a sub-event does not occur at that time. This step significantly enhances the model’s ability to perceive Weibo posts in terms of temporal distribution. Therefore, we incorporate the temporal features into the model with the expectation of improving the performance of the model on the Weibo classification task. Since the length of the temporal label is 31, this paper requires a network structure that can handle this dimension effectively. In this paper, we choose to use a simple but effective multilayer perceptron for feature extraction

To comprehensively capture the temporal information of Weibos, this study introduces a crucial temporal feature during model construction: the set of Weibo postings at each time node. Each post is assigned a timing label indicating whether its release time falls within the defined time period of each sub-event. “1" denotes the occurrence of a sub-event at that time, while a “0" indicates its absence. This step significantly enhances the model’s ability to perceive temporal distribution of Weibo posts. Consequently, temporal features are integrated into the model to enhance its performance on Weibo classification tasks. Specifically, a simple yet effective multilayer perceptron is employed for feature extraction. Temporal labels serve as inputs, and the MLP’s multilayer structure learns and captures the complex relationships between labels, as shown in Eq 12. The hidden layer of the MLP acts as a feature extractor, facilitating the extraction of higher-level abstract features from token labels. This approach not only enhances the model’s adaptability but also enriches temporal information in the deep learning model, thereby improving its performance on classification tasks.

time_embedding=MLP(time_labels) (12)

#### Feature fusion.

After semantic feature extraction and temporal feature extraction, this paper obtains text vectors and temporal vectors respectively, and by splicing these two kinds of key information, where ‘concat’ signifies the operation of vector concatenation, joining the text embedding and time embedding end-to-end to form a single, longer vector, this paper achieves a comprehensive correlation between the text content and the event temporal sequence. Subsequently, using the attention mechanism, this paper is able to selectively focus on different parts of the spliced vectors to more accurately capture features related to event classification.

combined_embedding=concat(text_embedding,time_embedding) (13)

attn_output=Attention(combined_embedding) (14)

The introduction of the attention mechanism enables the model to more accurately focus on different parts of the input features before making predictions, thus enabling it to adapt more flexibly to the relationships and weights between different input sources. This enables the model to be more targeted in capturing potential correlations between textual and time-series information, thereby improving the accuracy of feature representations.

## Experiment

### Analysis and presentation of experiments in story line construction

This section focuses on each step in the construction of the event story line, modifying various experimental parameters and demonstrating the final experimental results, i.e. the story line directed graph.

#### Model parameters.

It can be observed that the behaviour of ‘like’ on Weibo is more prevalent than that of ‘comment’ and ‘repost’. Conversely, commenting and ‘repost’ are more indicative of the extent to which users are influenced by and attentive to the text. Accordingly, we set *α* as 0.4, *β* as 0.4 and *γ* as 0.2 in the ‘Text Noise Filtering Based on Topic Heating Index’. It is also observed that Weibo posts pertaining to significant events tend to generate considerable social media engagement. Consequently, this paper sets the topic heat index threshold to the top 10% in order to filter out Weibo texts that have attracted a relatively large social media audience. This strategy enables the focus on Weibo that have attracted significant attention on social media, with the extraction of representative topic event vectors from them. This ensures that the constructed event vectors are more vivid and influential, while also improving the quality of the dataset.

#### Data processing.

This paper explicitly selected the top 10% of the topic hotness ranked Weibo text as the event story line corpus, resulting in a dataset comprising 932 Weibo posts and 903 hashtag tags. However, the direct input of these extensive hashtag collections into the algorithm would necessitate a significant investment of time and computational resources. In light of the pivotal role of hashtags in representing sub-events within the context of an event, this paper offers a comprehensive analysis of the data. Following the study and analysis, it was found that sub-events are less likely to persist throughout the course of a topic, but more likely to focus and trigger discussion during a specific time period. Based on this observation, in order to process the data more efficiently and save computational resources, this paper carries out further preprocessing of the dataset. The specific processing steps are as follows:

(1) Obtaining the time series of hashtag postings: This paper obtains the time series of hashtag postings by counting whether there are Weibo texts with specific hashtag labels each day within the crawled time range. Specifically, binary notation is used, where 1 indicates the existence of Weibo texts with the hashtag on that day, while 0 indicates otherwise.
(2) Filtering hashtag tags whose frequency of occurrence is too low: In this step, the number of occurrences of each hashtag in the entire dataset is counted and a frequency threshold is set. Specifically, only hashtag tags whose occurrences do not fall below the threshold are retained, while those with fewer occurrences and weaker relevance are excluded. This selective retention of high-frequency hashtags helps to focus the analysis on topics that have really attracted attention and discussion on social media, and provides more targeted data to support the subsequent construction of event vectors.
(3) Filtering out hashtag labels with excessive posting densities: In this step, the paper calculates the posting density of each hashtag in detail using its posting time series. By setting appropriate density thresholds, hashtags with excessively high posting densities, which may be related to multiple events or too general, are filtered out, while those with specific event associations are retained. For example, as shown in Fig ??, the hashtag ‘Japanese nuclear waste’ appears frequently on the timeline, and as a phrase it does not clearly express a single event. The aim of this step is to optimise the collection of hashtags to make them more suitable for constructing event contexts.

#### Evaluation indicators.

As the quality of event story line construction is difficult to clearly measure using traditional evaluation indicators, this paper relies more on intuitive observation to assess its quality. Compared to other fields or tasks, event story line construction involves multiple steps and complex data processing, making it challenging to comprehensively evaluate using a single quantitative indicator. Instead, this paper judges the quality of construction by observing the formation of event story line graphs, the presentation of node relationships, and the logical coherence of the overall structure. This intuitive observation approach emphasizes understanding the overall expression of event contexts, providing researchers with a comprehensive and intuitive evaluation method to better understand and interpret the evolution of event story line. Through this approach, the quality and effectiveness of event story line construction can be better assessed, leading to further optimization and improvement of the construction process. Therefore, intuitive observation plays an important role in evaluating event story line construction, providing researchers with valuable feedback and guidance.

#### Hashtag occurrence frequency threshold setting.

By setting different occurrence frequency thresholds, different sizes of event story lines can be obtained. Table 2 demonstrates the screening effect on Hashtag under different frequency thresholds and the number of final event story line nodes. As the frequency threshold increases, the number of Hashtags after frequency screening decreases significantly, indicating that higher frequency thresholds limit the selection of Hashtags.

**Table 2:** Number of hashtags with frequency thresholds.

frequency threshold	Number of Hashtags after frequency filtering	Number of Hashtags after text filtering	Number of story line nodes
1	878	528	268
2	223	154	111
3	107	80	70
4	71	55	49
5	50	39	37
6	42	33	31
7	29	23	22
8	19	16	16
9	12	10	10
10	7	6	6
11	4	4	4
12	3	3	3

It can be seen that the vast majority of Hashtag tags appear no more than 4 times, however, too large a frequency threshold will also lead to too few remaining Hashtags, thus failing to completely represent the event lineage, so a more appropriate frequency threshold ranges from 4 to 9.

**Fig 6:**
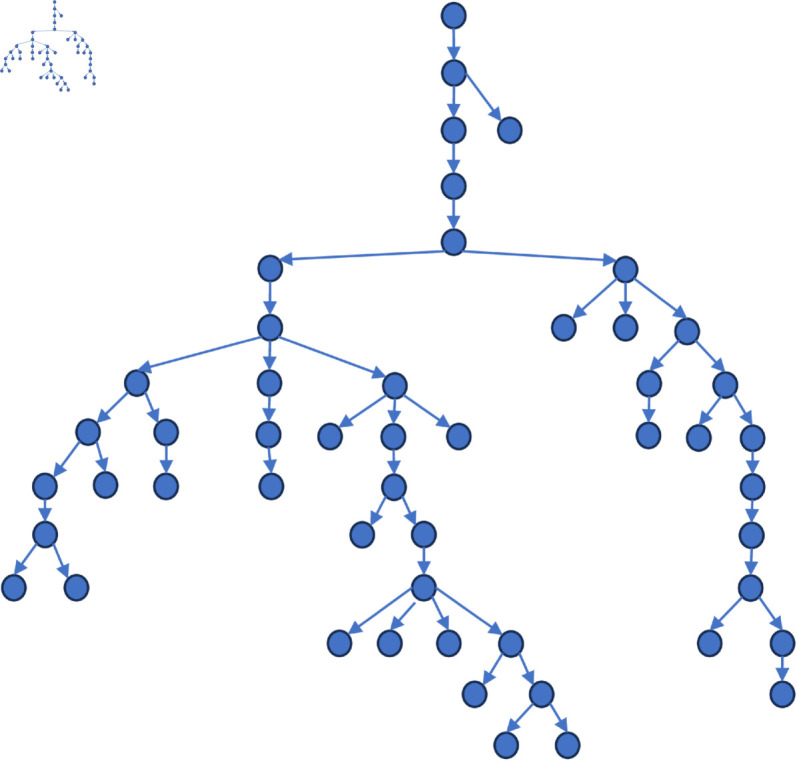
Demonstration of the structure of the story line diagram at different frequencies when threshold=4.

Fig 6 to Fig 11 shows the events obtained when the frequency threshold is 4 to 9 in the form of a tree diagram. According to the figure, when the frequency threshold is set to 6, the number of hashtags after frequency filtering is 42, the number of hashtags after text filtering is 33, and the number of nodes of the event story line constructed in the end is 31, which is a more desirable effect in balancing the information richness and structural simplicity.

**Fig 7:**
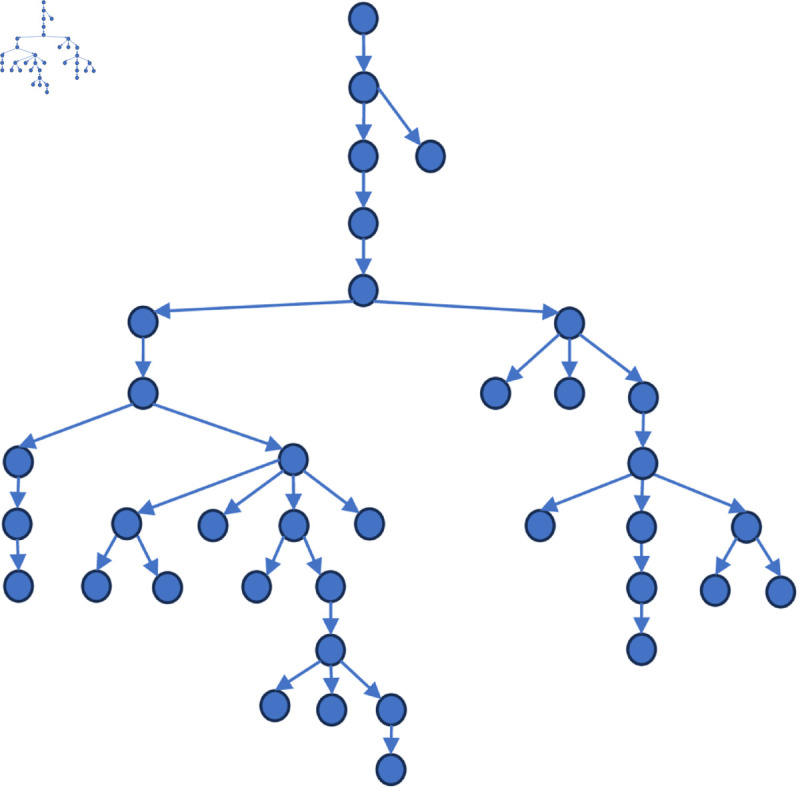
Demonstration of the structure of the story line diagram at different frequencies when threshold=5.

**Fig 8:**
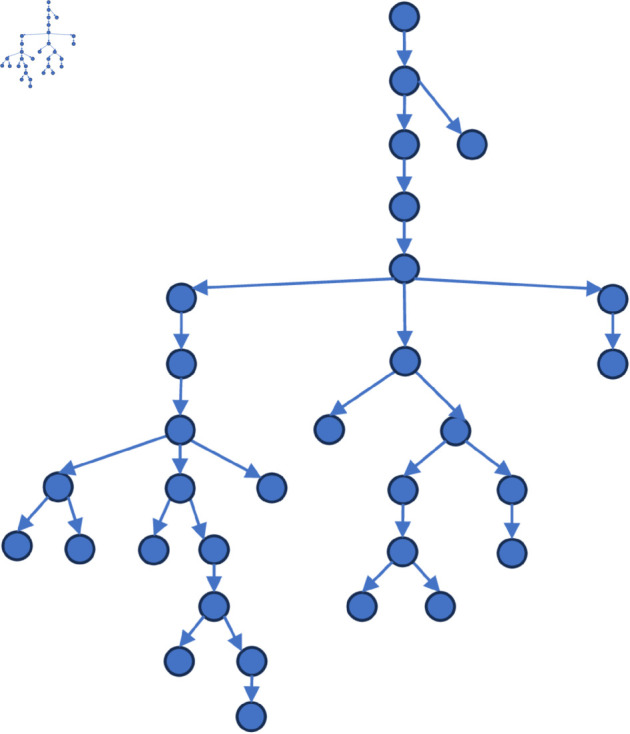
Demonstration of the structure of the story line diagram at different frequencies when threshold=6.

**Fig 9:**
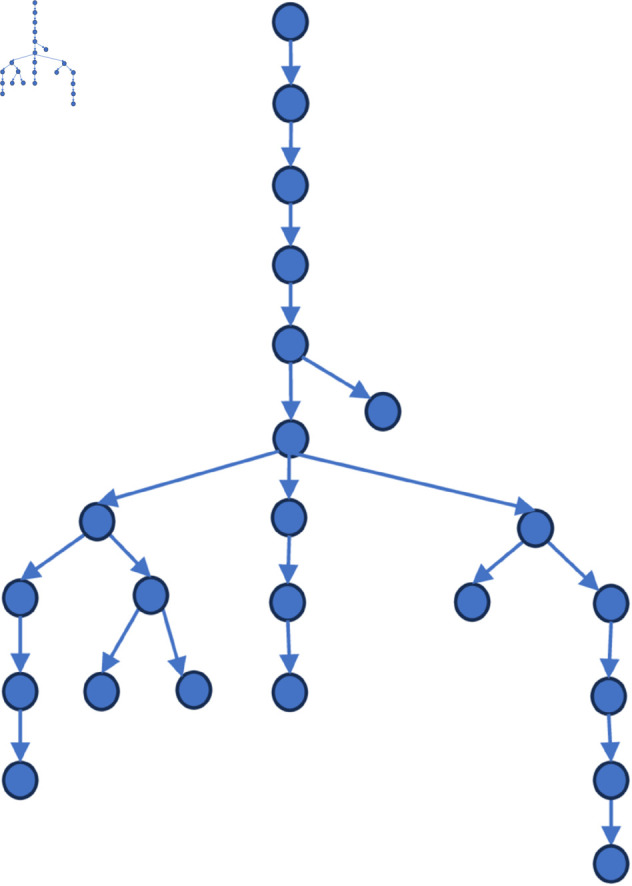
Demonstration of the structure of the story line diagram at different frequencies when threshold=7.

**Fig 10:**
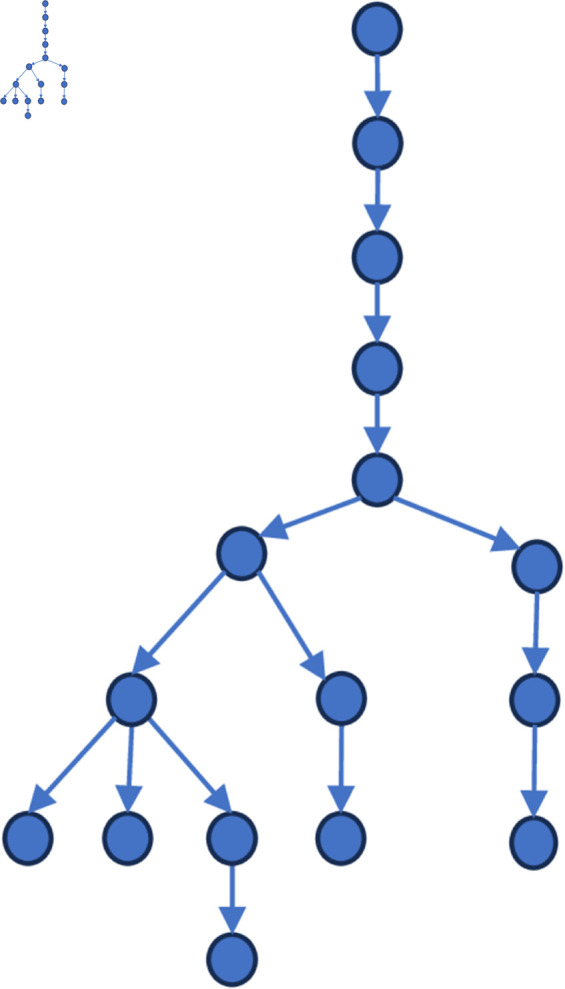
Demonstration of the structure of the story line diagram at different frequencies when threshold=8.

**Fig 11:**
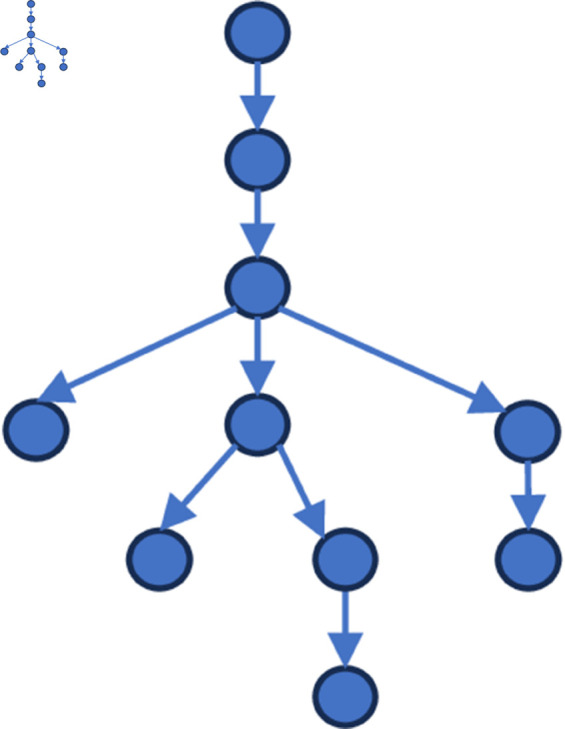
Demonstration of the structure of the story line diagram at different frequencies when threshold=9.

#### Density threshold setting.

Table 3 shows the effect of hashtag filtering under different density thresholds and the number of final event line nodes. As the density threshold increases, the number of density-screened hashtags gradually increases, suggesting that lower density thresholds allow more hashtags to be retained. At the same time, the number of event story line nodes constructed in the end shows a gradual increase, highlighting the significant effect of density thresholds on the construction of the event story line. When choosing the density threshold, it is necessary to strike a balance between the number of retained hashtags and the complexity of the event story line in order to satisfy the specific research purpose and needs. As this paper aims to cover as many important events of the topic as possible, the density threshold is set at 0.25 to ensure that the retained hashtags have a certain density but are not too large to construct a relevant and concise event line.

**Table 3:** Hashtag number varies with density thresholds.

Density Threshold	Hashtags after Density Filtering	Hashtags after Text Filtering	Nodes in the Network
0.1	26	24	23
0.15	33	28	27
0.2	37	30	29
0.25	42	33	31
0.3	46	37	33
0.35	48	38	34
0.4	49	39	35
0.45	52	41	37
0.5	53	42	38
0.6	57	43	38
0.7	59	44	37
0.8	60	44	37

#### Semantic similarity thresholds.

Table 4 shows the screening effect on hashtags under different text similarity threshold conditions and the number of final story line nodes. As the text similarity threshold increases, the number of hashtags screened gradually decreases, indicating that a higher text similarity threshold limits the selection of hashtags. The number of event story line nodes finally constructed also tends to gradually decrease, showing the significant effect of the text similarity threshold on event story line construction. Considering the quality of the hashtag and the complexity of the event story line, this paper chooses 0.6 as the best similarity threshold.

**Table 4:** Variation of hashtag count with text similarity threshold.

Text Similarity Threshold	Hashtags after Text Filtering	Nodes in the Network
0.4	37	35
0.45	37	35
0.5	35	33
0.55	34	32
0.6	33	31
0.65	27	25
0.7	17	17
0.75	6	6

#### Hashtag similarity formula parameter settings.

**Fig 12:**
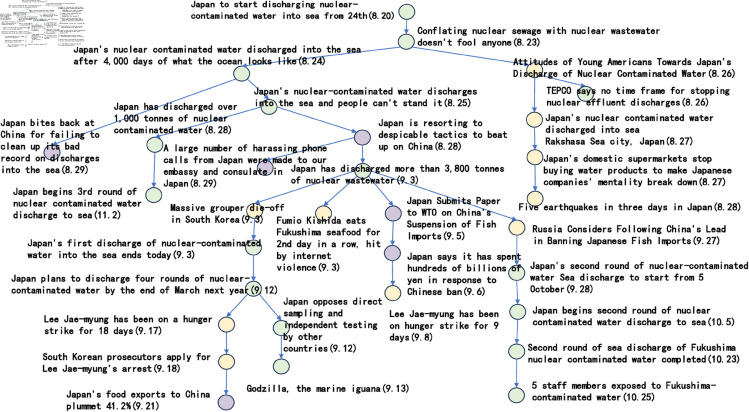
Presentation of the story line of “Japanese nuclear effluent” under different parameters when a=0.2, b=0.8.

After setting a frequency threshold of 6, a publication density threshold of 0.25, and a semantic similarity threshold of 0.6, we next discuss the parameter settings in the hashtag similarity calculation formula. Fig 12 to Fig 15 shows the branching of the story line under different parameter settings.

**Fig 13:**
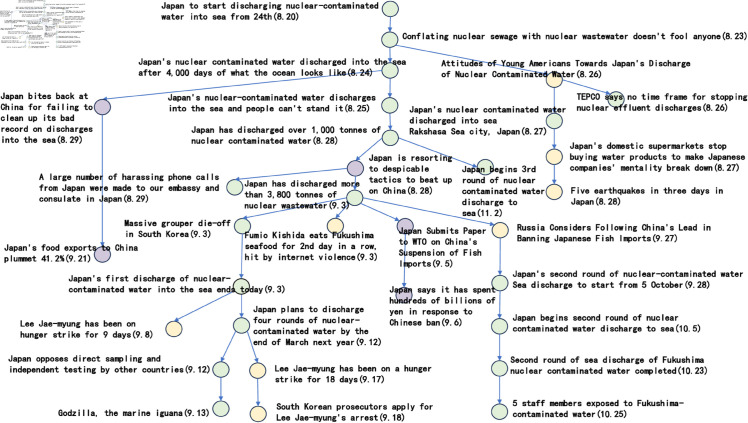
Presentation of the story line of “Japanese nuclear effluent” under different parameters when a=0.4, b=0.6.

**Fig 14:**
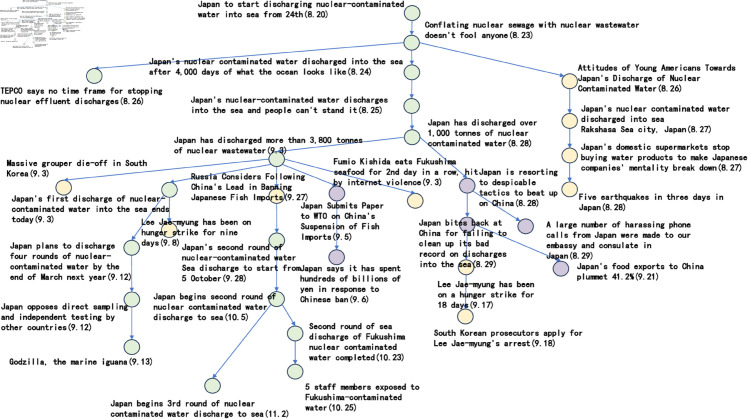
Presentation of the story line of “Japanese nuclear effluent” under different parameters when a=0.6, b=0.4.

**Fig 15:**
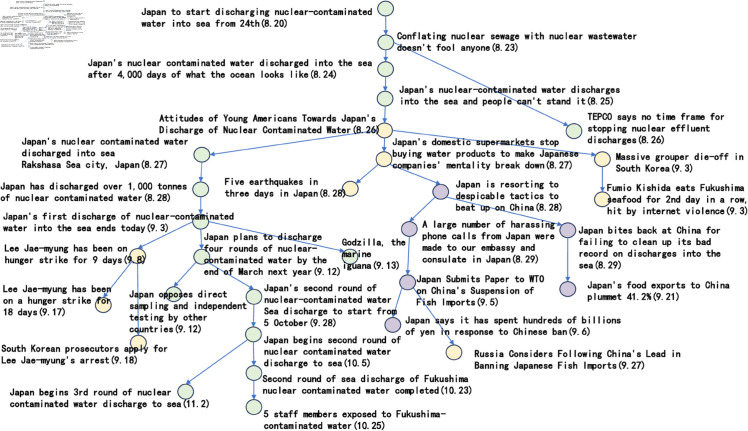
Presentation of the story line of “Japanese nuclear effluent” under different parameters when a=0.8, b=0.2.

The green nodes represent sub-events that are closely related to ‘Japanese nuclear leakage’, the yellow nodes represent other sub-events that are triggered by ‘Japanese nuclear leakage’, and the purple nodes represent sub-events that are closely related to China. Looking at the images, it can be seen that when the parameter a takes the value of 0.8 and b takes the value of 0.2, the subnodes of different colours are clustered more closely together. The implication of this phenomenon may be that the temporal feature, although significant in some cases, is not as directly effective as the textual content in reflecting the semantic association of labels.

#### Merge threshold settings.

The discussion proceeds to the parameter settings regarding the hashtag composite similarity threshold. Table 5 displays the count of nodes comprising event story lines under various similarity threshold conditions. As the merging threshold rises, the number of nodes gradually increases, suggesting that a higher merging threshold incorporates fewer hashtag nodes into the same event story line. Simultaneously, the count of nodes stabilizes, showing no significant rise as the merging threshold escalates to 0.85. From the table data, the count of nodes reaches 31 at a threshold of 0.8, thereafter trending towards stabilization. Consequently, this paper opts for 0.8 as the optimal integrated similarity threshold to strike a balance between maintaining the integrity of the event story line and mitigating redundant information.

**Table 5:** Variation of hashtag count with integrated similarity threshold.

Merging Threshold	Storyline Node Count
0.6	8
0.65	11
0.7	21
0.75	29
0.8	31
0.85	33
0.9	33

#### Tree diagram of story lines.

**Fig 16:**
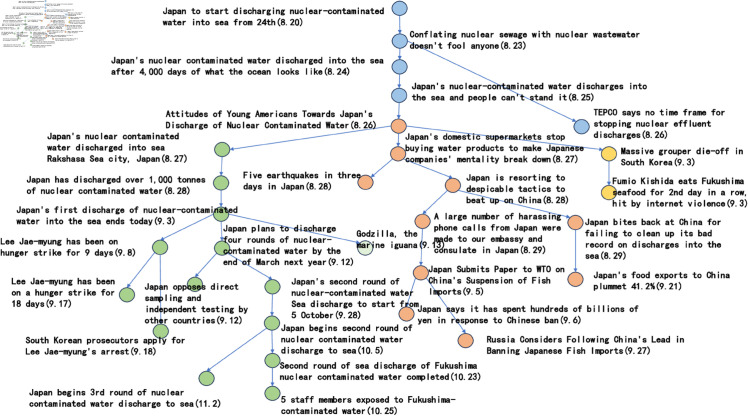
The story tree.

In the previous five subsections, this paper discusses in detail the parameter settings in the event story line construction step. Specifically, the frequency threshold is set to 6, the publication density threshold is 0.25, the semantic similarity threshold is 0.6, the similarity calculation parameter a takes the value of 0.8, b takes the value of 0.2, and the Hashtag composite similarity threshold is 0.8. By using these parameter settings, this paper successfully builds a story line diagram about the event of “Japan’s nuclear wastewater" and the event of “Japan’s nuclear wastewater". “event of the story line tree graph. The story nodes are shown in Table 6, and the story tree composed of these nodes is shown in Fig 16.

**Table 6:** Storyline nodes.

Node ID	Node Title
1	Japan to start discharging nuclear-contaminated water into sea from 24th
2	Conflating nuclear sewage with nuclear wastewater doesn’t fool anyone
3	Japan’s nuclear contaminated water discharged into the sea after 4,000 days of what the ocean looks like
4	Japan’s nuclear-contaminated water discharges into the sea and people can’t stand it
5	Attitudes of Young Americans Towards Japan’s discharge of Nuclear Contaminated Water
6	Japan’s nuclear contaminated water discharged into sea; Rakshasa Sea city, Japan
7	Japan’s domestic supermarkets stop buying water products to make Japanese companies’ mentality break down
8	TEPCO says no time frame for stopping nuclear effluent discharges
9	Five earthquakes in three days in Japan
10	Japan has discharged over 1,000 tonnes of nuclear contaminated water
11	Japan is resorting to despicable tactics to beat up on China
12	A large number of harassing phone calls from Japan were made to our embassy and consulate in Japan
13	Japan bites back at China for failing to clean up its bad record on discharges into the sea
14	Massive grouper die-off in South Korea
15	Japan’s first discharge of nuclear-contaminated water into the sea ends today
16	Fumio Kishida eats Fukushima seafood for 2nd day in a row, hit by internet violence
17	Japan submits paper to WTO on China’s suspension of fish imports
18	Japan says it has spent hundreds of billions of yen in response to Chinese ban
19	Lee Jae-myung has been on hunger strike for 9 days
20	Japan plans to discharge four rounds of nuclear-contaminated water by the end of March next year
21	Japan opposes direct sampling and independent testing by other countries
22	Godzilla, the marine iguana
23	Lee Jae-myung has been on a hunger strike for 18 days
24	South Korean prosecutors apply for Lee Jae-myung’s arrest
25	Japan’s food exports to China plummet 41.2%
26	Russia considers following China’s lead in banning Japanese fish imports
27	Japan’s second round of nuclear-contaminated water Sea discharge to start from 5 October
28	Japan begins second round of nuclear contaminated water discharge to sea
29	Second round of sea discharge of Fukushima nuclear contaminated water completed
30	5 staff members exposed to Fukushima-contaminated water
31	Japan begins 3rd round of nuclear contaminated water discharge to sea

**Fig 17:**
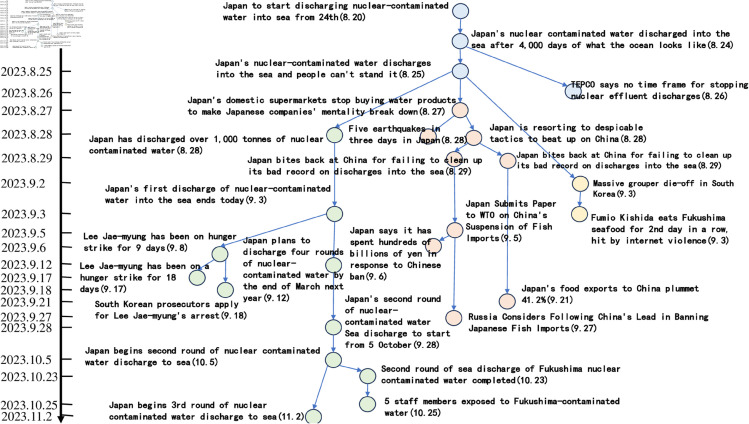
Tree diagram of the story line of the “Japanese nuclear effluent” incident.

Considering the clarity of the event representation, this paper further performed a manual pruning process on the resulting story line graph. In this process, the paper focuses on the removal of branches that may be ambiguous or redundant, and adds a timeline to the graph in order to more clearly observe the content development of events over time. After careful pruning and addition of timelines, the resulting story line diagram presents a clearer and more concise path of events, as shown in Fig 17. This optimised diagram will provide a more intuitive perspective and basis for subsequent analysis and understanding, which will help to further explore the process of event evolution as well as the related correlations.

Based on the story line, it can be observed that the ‘Japan Nuclear Sewage’ event has triggered discussions since 20 August 2023, and between 20 August 2023 and 29 August 2023, the nodes are intensive, showing that users pay high attention to the event and discuss it frequently. These discussions mainly focus on the impacts caused by the nuclear effluent discharge in Japan. In addition, the dendrogram clearly points out that from 25 August 2023 onwards, the overall event development is divided into three main directions. The green branch is the trunk with the largest number of nodes, through which it is possible to clearly understand the schedule of ‘Japan’s nuclear effluent’ releases, including the amount of discharges as well as the start and end dates of each round of releases. The orange node branch describes the political and economic impacts of the ‘Japanese nuclear effluent’ release. This branch shows that the ‘Japanese nuclear effluent’ discharges caused large negative political impacts and economic losses. In contrast, the yellow branch has fewer nodes and mainly shows other aspects of the news caused by the ‘Japanese nuclear effluent’, which are mostly negative.

### Validation of RoMLP-AttNet model validity

#### Model parameters.

**Fig 18:**
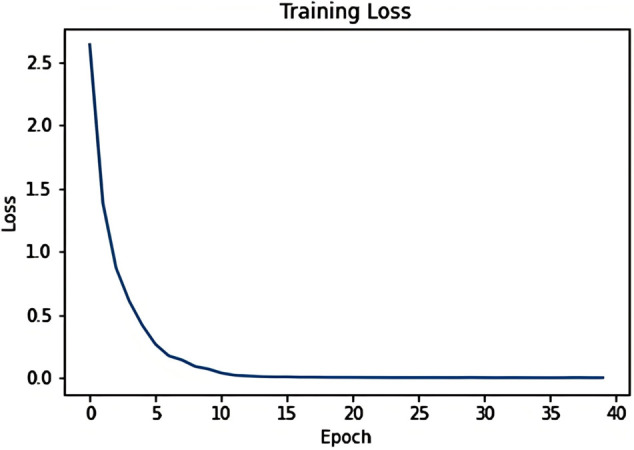
Change in loss ratio with rounds.

Tokenize participle filler length is set to 512, the model is optimised using adam algorithm, and the learning rate is set to 0.0001. We first set a larger number of training rounds, and get the change of loss rate with the number of rounds as shown in Fig 18, and according to the picture, it can be seen that when the number of training rounds reaches 15 or so, the loss rate tends to be stable, considering that the training should be as full as possible and prevent overfitting, this paper finally set the number of training rounds to 20.

#### Evaluation indicators.

In this experiment, precision rate, accuracy rate and F1 value are used as objective evaluation indexes.

(1) Precision (abbreviated as P) is for the prediction results, indicating the accuracy with which positive samples are correctly predicted, with larger P indicating fewer false detections.
  $$p=\frac{TP}{TP+FP}$$ (15)
(2) Recall (abbreviated as R) is a metric used to measure the performance of a classification model, which measures the model’s ability to identify positive example samples. Recall indicates the proportion of the number of samples correctly identified as positive examples by the model to the total number of positive examples.
  $$R=\frac{TP}{TP+FN}$$ (16)
(3) The F1 score considers both precision and recall, so that both are simultaneously maximised and balanced.
  $$F1=\frac{2\times p\times R}{TP+FP+TN+FN}$$ (17)

In the above formula, TP denotes the number of samples that are actually positive samples and correctly predicted, FP denotes the number of samples that are actually negative samples but incorrectly predicted as positive samples, TN denotes the number of samples that are actually negative samples and correctly predicted, and FN denotes the number of samples that are actually positive samples but incorrectly predicted as negative samples.

#### Validation of the effectiveness of the reduced dimensional network.

The RBT3 model is used as a benchmark for the extraction of features from Weibo text. Traditional pre-trained models like RBT3 often output high-dimensional features (768 dimensions for BERT-like models), which may introduce redundancy for short Weibo texts (average 140 words), leading to overfitting and inefficient computation. To address this, we introduces a dimensionality reduction network after the output of the RBT3 model to extract Weibo text features. In this process, this paper disregards the time series features and instead classifies the text features directly. Given that the input dimension of the dimensionality reduction network is 768, it follows that the dimensionality of the output of the dimensionality reduction network must be less than 768. The output dimensions K are set to 64, 128, 256, 384 and 512, respectively, and the classification results under these different dimensions are evaluated. The specific evaluation results are presented in Table 7.

**Table 7:** Evaluation metrics for different output dimensions of dimension reduction networks.

Evaluation Metric	RBT3	RBT3+Reduce(64)	RBT3+Reduce(128)
R	0.555	0.5602	0.6021
P	0.6328	0.6354	0.6808
F1	0.5645	0.5729	0.6209
**Evaluation Metric**	**RBT3+ Reduce(256)**	**RBT3+Reduce(384)**	**RBT3+Reduce(512)**
R	0.5759	0.5916	0.5759
P	0.6193	0.6379	0.6636
F1	0.5783	0.5908	0.5926

According to Table 7, it can be seen that all the model classifications with the addition of the dimensionality reduction network are higher than the model classifications with only RBT3 as the feature extraction. Different output dimensions show differences in accuracy, precision and F1 score. When the output dimension is 128, the model achieves a more desirable result in terms of overall performance, with relatively high accuracy, precision and also balanced in terms of F1 score.

#### Validation of the effectiveness of temporal feature extraction.

On the basis of text features, this paper introduces time series to further enrich Weibo information.Existing event detection methods often overlook explicit temporal modeling, treating time as implicit context in sequences, which fails to capture the chronological order and periodicity of events. The time series features are extracted by MLP network and the text features are spliced with the time features for classification. In order to verify the validity and importance of the temporal features, this paper compares the model with MLP network added with the model with only the dimensionality reduction network added. According to the previous section, this paper chooses the dimensionality reduction model with an output dimension of 128 as the benchmark, and then sets the output dimensions of MLP to 16, 32, 64, 128 and 256 respectively to get the classification results of each dimension.

RoMLP-AttNet’s explicit temporal feature extraction (via MLP) addresses a key limitation of traditional sequence models: the inability to model “when an event occurs" as a standalone feature. According to Table 8, it can be seen that after adding the time series features, the model classification results are significantly improved with at least 17.28% increase in recall, at least 12% increase in precision, and at least 15.2% increase in F1 value. Different MLP output dimensions have a significant impact on the performance of the Weibo classification model.RBT3+Reduce+MLP(128) performs the best in terms of recall, precision, and F1 scores, reaching 0.8325, 0.8531, and 0.8315, respectively.The performance of the model gradually improves with the increase of the MLP output dimensions.However, RBT3+ Reduce+MLP(128) presents a relatively balanced state in terms of comprehensive performance. Therefore, for the Weibo classification task, choosing the configuration with MLP output dimension of 128 can obtain the optimal performance performance with high recall, precision and F1 value, which provides effective support for the reliability of the model in practical applications. This demonstrates that temporal-semantic joint modeling is indispensable for Weibo event detection, as it resolves ambiguities between semantically similar but temporally distinct events, a capability lacking in existing methods.

**Table 8:** Evaluation metrics for different output dimensions of MLP.

Evaluation Metric	RBT3+ Reduce	RBT3+Reduce +MLP(16)	RBT3+Reduce +MLP(32)
A	0.6021	0.7749	0.7853
P	0.6808	0.8008	0.8289
F1	0.6209	0.7729	0.7945
**Evaluation Metric**	**RBT3+Reduce +MLP(64)**	**RBT3+Reduce +MLP(128)**	**RBT3+Reduce +MLP(256)**
A	0.8063	0.8325	0.8220
P	0.8090	0.8531	0.8302
F1	0.7973	0.8315	0.8153

#### Comparative experiments.

We choose the following models as comparison models:

(1) FastText+CNN model: using FastText as a pre-training model, Umer [50] proposed a CNN-based model for short and long text classification of sequences for the text classification task, and experimented on seven datasets, achieving state-of-the-art results on five. Therefore, it is chosen as one of the comparison models . In the comparison experiments, this paper sets one set of inputs as Weibo text and another set of inputs as splicing of Weibo text with time series.
(2) RBT3-Attention: in order to verify the effectiveness and sophistication of this paper in extracting textual features and temporal features respectively, and the inputs are the splicing of text and time series.
(3) TextBiLSTM+Attention: the Att-BLSTM model proposed by Wang [?] utilises the neural attention mechanism in combination with a bidirectional long and short-term memory network (BLSTM) in order to capture the most important semantic information in a sentence. In this paper, this classical text classification model is chosen as one of the comparison models. In the comparison experiments, this paper sets one set of inputs as Weibo text, and the other set of inputs as the splicing of Weibo text with time series.

We train three models separately using the constructed categorical dataset and show the obtained results in Table 9.

**Table 9:** Text classification results for different methods.

Evaluation Metric	RoMLP-AttNet	FastText+CNN^1^	FastText+CNN^2^
R	0.8325	0.8168	0.5916
P	0.8531	0.8187	0.6287
F1	0.8315	0.8067	0.5902
**Evaluation Metric**	**RBT3-Attention**	**TextBiLSTM +Attention^1^**	**TextBiLSTM +Attention^2^**
R	0.8272	0.7696	0.5602
P	0.8296	0.7901	0.6158
F1	0.821	0.7635	0.5597

The superscript 1 indicates that the input is the splicing of Weibo text with time series, and the superscript 2 indicates that the input is the Weibo text. Table 9 shows that the models proposed in this paper demonstrate superior performance in the text classification task. When the input of other models contains only Weibo text, the RoMLP-AttNet model proposed in this paper outperforms the other compared models in recall, precision, and F1 value, with average improvements of 21.73%, 18.22%, and 21.02%, respectively. When the input is text with time series, the model proposed in this paper outperforms the other comparative models in terms of classification results, improving recall by at least 0.53%, precision by at least 2.35%, and F1 value by at least 1.41%.

## Conclusion

In this study, we proposes an integrated framework for event detection and storyline construction on Weibo, combining temporal dynamics, hashtag semantics, and deep learning techniques. Our contributions lie in three key advancements: (1) a temporal-semantic fusion method that enhances event correlation analysis by jointly modeling hashtag release sequences and semantic similarity; (2) a timeline-based merging algorithm that hierarchically organizes hashtags into coherent storylines; (3) the RoMLP-AttNet model, which achieves significant performance gains (16.73% recall, 15.8% precision) by fusing text and temporal features. These innovations address challenges in handling noisy, fragmented social media data while maintaining interpretability.

While these innovations effectively address challenges in handling noisy, fragmented social media data, certain limitations merit consideration. For instance, the model’s performance may diminish for low-visibility events with sparse data, where temporal patterns and hashtag densities are less pronounced. Additionally, the framework’s dependency on Weibo-specific features—such as hashtag usage norms and interaction metrics could limit its direct applicability to platforms like Twitter or TikTok, which exhibit distinct user behaviors. Cross-lingual generalization remains another challenge, as the semantic and cultural nuances of Chinese hashtags may not translate seamlessly to other languages. Future efforts will focus on enhancing adaptability through lightweight fine-tuning strategies for small-scale events, cross-platform feature alignment, and multilingual embeddings to broaden cultural and linguistic coverage.

Practically, the framework enables real-time monitoring of public opinion, crisis management, and business intelligence. Future work will refine scalability and cross-platform adaptability to broaden its utility in global contexts.This study advances social media analytics, offering actionable insights for policymakers, enterprises, and researchers to navigate dynamic information landscapes.
